# Vertebrobasilar Contribution to Cerebral Arterial System of Dromedary Camels (*Camelus dromedarius*)

**DOI:** 10.3389/fvets.2021.696707

**Published:** 2021-06-11

**Authors:** Ahmad Al Aiyan, Preetha Menon, Adnan AlDarwich, Moneeb Qablan, Maha Hammoud, Turke Shawaf, Ken Richardson

**Affiliations:** ^1^Department of Veterinary Medicine, College of Food and Agriculture, United Arab Emirates University, Al Ain, United Arab Emirates; ^2^Department of Clinical Sciences, College of Veterinary Medicine, King Faisal University, Al-Hasa, Saudi Arabia; ^3^College of Veterinary Medicine, School of Veterinary and Life Sciences, Murdoch University, Perth, WA, Australia

**Keywords:** brain, circle of Willis, corrosion cast, dromedary, camel, vertebrobasilar system, carotid system

## Abstract

It is hypothesized that in the “more highly evolved” mammals, including the domesticated mammals, that the brainstem and the cerebellum receive arterial blood through the vertebrobasilar system whilst the internal carotid arteries primarily supply the forebrain. In camels, the arterial blood supply to the brain differs from that of ruminants since the internal carotid artery and the rostral epidural rete mirabile (RERM) are both present and the basilar artery contributes a significant proportion of cerebral afferent blood. In this study, we described the anatomical distribution of the vertebrobasilar system arterial supply in the dromedary. Secondly, we determined the direction of blood flow within the vertebral and basilar arteries using transcranial color doppler ultrasonography. Thirdly, we quantified the percentage arterial contributions of the carotid and vertebrobasilar systems to the dromedary brain. Fifty-five heads of freshly slaughtered male Omani dromedaries aged 2–6 years were dissected to determine the distribution and topography of the arterial distribution to the brain. Their anatomical orientation was assessed by casting techniques using epoxy resin, polyurethane resin and latex neoprene. The epoxy resin and polyurethane resin casts of the head and neck arteries were used to measure the diameter of vertebrobasilar arterial system and carotid arterial system at pre-determined locations. These arterial diameters were used to calculate the percentage of blood supplied by each arterial system. The vertebrobasilar system in dromedary camels consists of paired vertebral arteries that contribute to the ventral spinal artery and basilar artery at multiple locations. In most specimens the vertebral artery was the primary contributor to the basilar artery compared to that of the ventral spinal artery. In four specimens the ventral spinal arteries appear to be the dominant contributor to the basilar artery. Transcranial color doppler ultrasonography confirmed that the direction of blood flow within the vertebral and basilar arteries was toward the brain in animals examined in ventral recumbency and when standing. The vertebrobasilar system contributes 34% of the blood supply to the brain. The vertebrobasilar system is the exclusive supply to the medulla oblongata, pons and cerebellum.

## Introduction

In mammals, the high metabolic demands of neurons have resulted in the central nervous system having an acute dependence on a constant supply of oxygenated blood. Depending on species, at any single time interval the brain uses about 18% of total blood volume (1, 2). The brain's high oxygen and glucose consumption rates are disproportionate to its mass. Despite its high metabolic turnover, it does not have back up reserves to augment its nutritional demands. Consequently, the brain depends on an unvarying elevated supply of arterial blood for its ongoing sustenance (3, 4). Researchers have identified a series of gradual evolutionary changes in the cranial afferent vasculature architecture, notably the addition of the vertebral arteries to supply the brainstem and the cerebellum. These changes have been observed during the evolutionary transition from non-mammalian vertebrates to mammals. The primitive mammals, i.e., those having a dominant olfactory forebrain and basic life supporting hindbrain, depend solely on the internal carotid artery for their cerebral arterial blood. As the mammalian brain evolved into a larger and more complex entity the hypothesized “mammalian shift” in its vascular supply is most likely due to the need for additional sources of oxygenated blood to the brain. The increased contribution of the vertebrobasilar system parallels the evolutionary expanding cerebellum and cerebellar peduncles (5–8). The vertebral arteries with their increased contribution to the brain has been documented in canids and equids (6, 9). Though all mammals and non-mammalian vertebrates have a basilar arterial supply, the blood supply to the brain through the basilar artery is seen to increase with the evolution of more highly complex brains (7).

In non-mammalian vertebrates the brain is supplied solely by a pair of internal carotid arteries anastomosing with a cerebral arterial circle. It is hypothesized that in the “more highly evolved” mammals, including the domesticated mammals, that the paired internal carotid arteries are inadequate in the provision of a satisfactory arterial blood supply to both the enlarged forebrain and the hindbrain (10, 11). Hence in domesticated mammals the development of the vertebrobasilar system supplying the brainstem and the cerebellum whilst the internal carotid arteries primarily supply the forebrain (10). In both sheep and cattle, the blood flow to the brain through the carotids is significantly higher than that of the vertebral arteries. In sheep, the carotid arterial system is the principal afferent blood supply to the cerebral arterial circle whilst the basilar artery primarily acts as an efferent channel transporting arterial blood from the cerebral arterial circle to supply the medulla oblongata and cervical region of the spinal cord (12–16). Contrastingly the basilar artery acts as an afferent channel to the cerebral arterial circle in the horse and dog where it has a greater role in supplying the brainstem and the cerebellum (6, 9).

In camels, the arterial blood supply to the brain differs from that of ruminants since the internal carotid artery and the rostral epidural rete mirabile (RERM) are both present and the basilar artery contributes a significant proportion of cerebral afferent blood (17).

While many studies have described the arterial supply to the dromedary brain in a qualitative manner (10, 18–22), none have quantified the arterial contribution of the internal carotid and vertebrobasilar systems. One study reports that in dromedaries the percentage contribution of the maxillary artery, internal carotid artery and external ophthalmic artery to the RERM are 76.41, 13.33, and 10.26%, respectively (23). However, this study ignores the middle meningeal artery which has been proven subsequently to contribute to the RERM (17). Likewise they didn't measure the contribution of the vertebrobasilar system to the brain.

In this study, we describe the anatomical distribution of the vertebrobasilar system arterial supply in the dromedary. Secondly we quantifythe percentage arterial contributions of the carotid and vertebrobasilar systems to the dromedary brain. Thirdly we determine the direction of blood flow within the vertebral and basilar arteries.

## Materials and Methods

This study adhered to the Research Ethics Policy and was approved by the Animal Research Ethics Committee at the United Arab Emirates University (ERA_2019_5850). The reported experiments comply with the Animal Research: Reporting of *In vivo* Experiments Guidelines of the United Arab Emirates University.

In this study, fifty-five heads of freshly slaughtered male Omani dromedaries aged between 2 and 6 years were procured from Al Khazna, Abu Dhabi Food Control Authority (ADFCA) and Bawadi, Al Ain city municipality Camel Slaughterhouses and dissected to determine the distribution and topography of the arterial distribution to the brain.

The heads all retained a portion of the neck that included several vertebrae, ranging variably from the third to the seventh cervical vertebrae. At the exposed end of the neck the right and left common carotid arteries were cannulated and between 400 and 800 mL of casting agent injected slowly under hand pressure. In this study three different red colored solutions were used as casting agents, two of them are hard casts, an epoxy resin and a polyurethane resin plus a soft cast i.e., latex neoprene. Thirty heads were injected with red epoxy resin (Gulfguard Epoxy—Color 04E53) mixed with the solvent-free hardener (Falco Epoxy Hardener) made up in a ratio of 4:1, just before injection. Twenty heads were injected with a liquid polyurethane resin (Polytek EasyFlo 60 Liquid Plastic) mixed with its hardener in the ratio 1:1, just before injection. The remaining five camel heads were injected with red colored latex neoprene (Latex Globalsil AL 20, GlobalChemica S.R.I) mixed with an acid hardener in the ratio 10:1, just before injection.

The smaller heads required ~400 mL and the larger heads ~600 mL of casting agent. Samples having seven cervical vertebrae required ~800 mL of casting agent. The solutions were injected slowly with hand pressure, using 60-mL syringes, until resistance was felt. To prevent the leakage of the casting agent out of anastomosing vessels, such as the vertebral and spinal arteries, they were clamped with artery forceps. Once perfusion was complete the heads were then held at 5°C for a minimum of 24 h, until the arteries were palpably hard.

The skin and muscles of the heads were dissected and removed. The roof of the cranium and vertebrae of all samples were sliced open with a rotating power saw (DeWalt DWE4001; DeWalt 100X0.1X16 mm blade). The following landmarks were used for this exercise; laterally the level immediately dorsal to the zygomatic arch, rostrally the connection between the right and left temporal line and caudally above the occipital condyles.

In twenty heads the cranial cavity was flushed using a high-pressure water jet. This removed all of the brain tissues and spinal cord without affecting the epoxy or polyurethane casts. Ten craniotomized heads together with their first two spinal vertebrae were immersed in a 5 percent potassium hydroxide (Caustic Potash-solid 90%, Albemarle, Jordan) that digested all soft tissues as well as bone but not the epoxy or polyurethane in the arteries. This process took 2 weeks and resulted in “stand alone” 3-dimensional vascular casts of the arterial supply to the brain and upper spinal cord. Twenty heads (thirteen with the first two cervical vertebrae present and seven with all seven cervical vertebrae present) were digested in a sodium carbonate solution at 60°C for 3–5 days, before high pressure water flushing of the soft tissues away from the nearby bones. The resultant casts displayed the course of the vertebrobasilar arterial system from cervical vertebra seven through to the floor of the cranium.

The five latex-injected camel heads were immersed in 6% formaldehyde for a week and then the brain together with its attendant arteries was dissected free and extracted from the cranial cavity to study the blood supply to different regions of the brain.

### Identifying Direction of Blood Flow in the Basilar Artery

**Figure 1:**
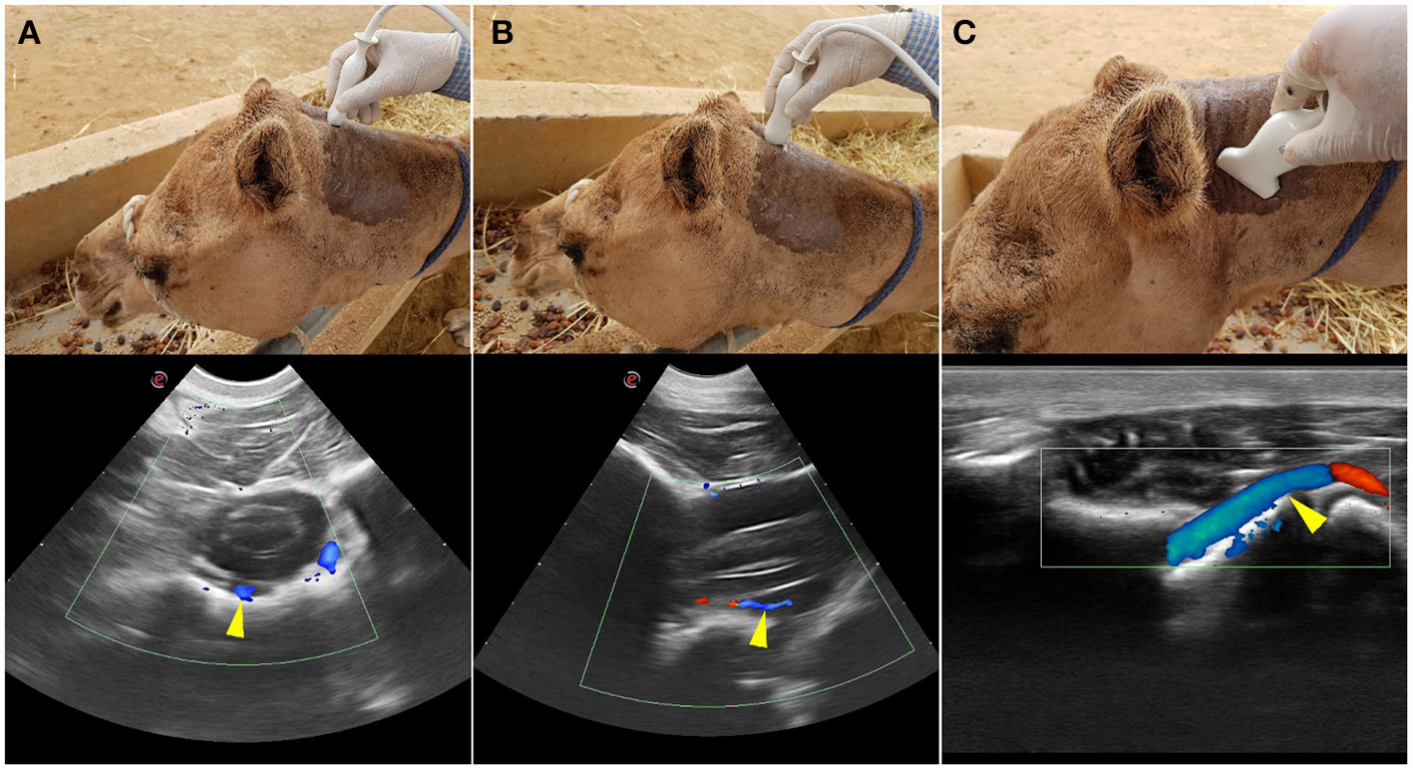
Transcranial Color Doppler flow images at the atlanto-occipital window where **(A)** transverse plane and **(B)** sagittal plane. Here the basilar artery is blue (arrowed) and the two red spots are the double root of the caudal cerebellar artery; **(C)** transverse plane at the level between the first and the second cervical vertebrae. Here blue (arrowed) is the vertebral artery with blood flow toward the brain. The red highlighted area of the vertebral artery indicates the blood flow toward the transducer due to the curved route of the vertebral artery in this area. Red indicated blood flow toward the transducer and blue is away from the transducer.

**Figure 2:**
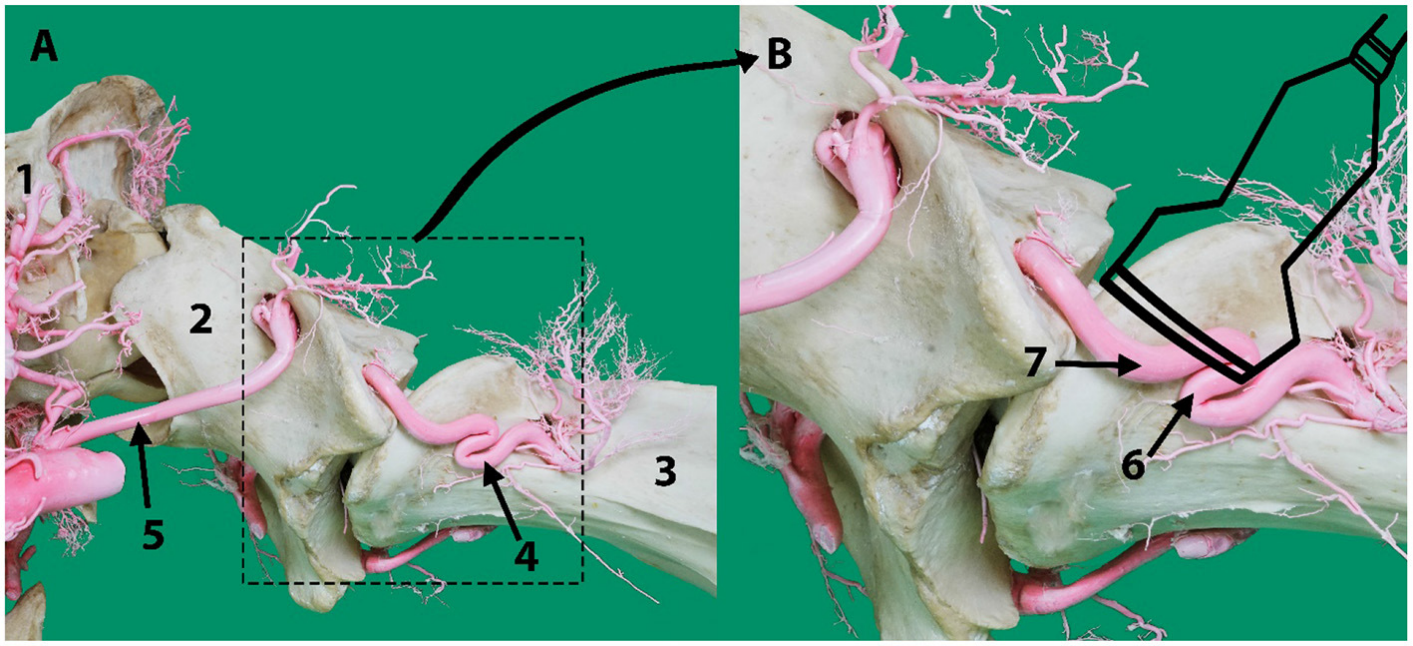
**(A)** Lateral aspect of an arterial resin cast showing the external route of the vertebral artery relative to cervical vertebrae C1 (atlas) and C2 (axis). **(B)** An outline of the transducer probe is superimposed over the location of the vertebral artery to explain the presence of the two colors in Figure 1C indicating the blood flow toward (6) and away (7) from the transducer due to the curved route of the vertebral artery in this area. Here 1, skull; 2, atlas; 3, axis; 4, vertebral artery; 5, occipital artery.

To identify the direction of blood flow in the basilar artery, transcranial color doppler ultrasonography (Esaote MyLabDelta, SC3123 Micro Convex Probe, 4.2 MHz) was performed on two live camels. The transducer was placed at the atlanto-occipital space just below the occipital protuberance and oriented toward the camel's muzzle (Figures 1A,B). In the median plane view, the basilar artery immediately ventral to the medulla oblongata was captured to determine its direction of blood flow. This was repeated in ventral recumbency as well as in a normal standing position. The direction of blood flow was assessed with the neck in both extended and hyperflexion positions. The same process was repeated in the sagittal plane at the level between the first and the second cervical vertebrae to identify the direction of blood flow within the vertebral artery (Figures 1C, 2). The vertebral artery was coded blue indicating the direction of blood flow was toward the brain away from the transducer (Figure 1C).

### Measuring Cranial Arteries

**Figure 3:**
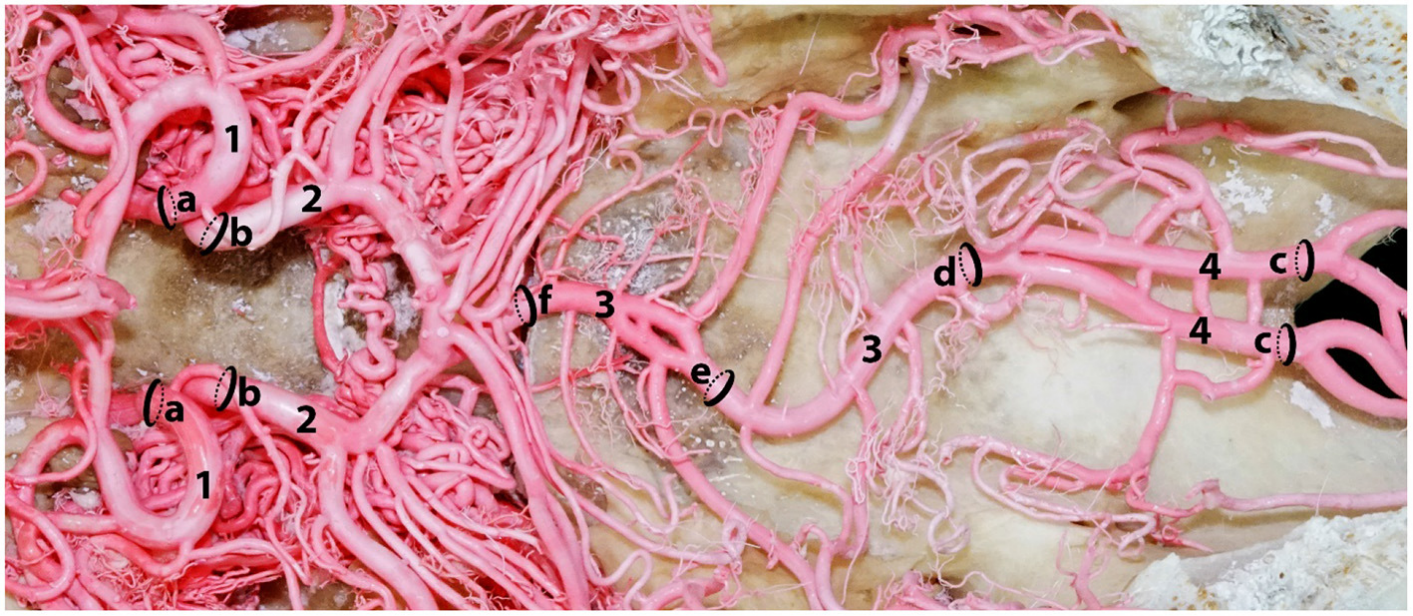
A dorsal photograph showing the measurement sites of the afferent arterial supply to the ventral aspect of the dromedary brain. Here; 1, rostral cerebral artery; 2, caudal communicating artery; 3, basilar artery; 4, right and left medullary segments of basilar artery. The measurement of a and b indicate the contribution of the carotid arterial system to the brain while c indicates the contribution of the vertebrobasilar system to the brain. Sequential diameter measurements of the basilar artery were taken at d, e and f.

A series of arterial diameters were measured using a digital Vernier caliper (Insize Digital Caliper -ISZ-1108-150) at pre-determined locations of the epoxy and polyurethane casts (Figure 3). Measurements were 2 mm away from the origin or anastomosing portions of the arteries. We excluded those arteries that had hemorrhaged from our calculations. To identify the carotid contribution to the cerebral circulation, the diameter of the rostral cerebral artery and caudal communication arteries were measured 2 mm away from their point of origin from the rostral epidural rete mirabile (Figure 3). To estimate the contribution of the vertebrobasilar system to the brain, measurements were taken 2 mm rostral to the point of anastomoses of the ventral spinal artery and medial branch of vertebral artery (Figure 3). These loci were selected to estimate the maximum volume of arterial blood emerging from the carotid and vertebrobasilar arterial systems, before they branched out supplying the brain.

The arterial diameters determined above were used in calculating the percentage blood supplied by vertebrobasilar arterial system and carotid arterial system. All calculations were done in MS Excel. We also estimated the variation between the arteries in the left and right side. All of the samples were imaged using Sony a7R II at 42-megapixel Fine JPEG setting for acquiring high-resolution images.

### Estimating the Percentage Contributions of the Carotid and Vertebrobasilar Systems

We estimated the percentage contribution of these two arterial systems to the brain (cerebrum, cerebellum and brain stem) by measuring the diameter of arteries that exclusively supply the brain in the epoxy and polyurethane casts of 28 animals. When determining the carotid system contribution we measured the arteries emerging from the RERM, namely the rostral cerebral artery and caudal communicating artery, because of their exclusive contribution to the brain. The point of measurement was 2 mm from their origin from the RERM, as shown in Figure 3.

To determine the vertebrobasilar contribution to the brain we measured the basilar artery at a point before it branches to supply the brain stem at the level of the occipital condyles where the medial branch of the vertebral artery fuses with the ventral spinal artery to form the medullary segment of the basilar artery (Figure 3). This location served as the best approximation of vertebrobasilar supply to the medulla oblongata, pons and cerebellum, before supplying the cerebrum through the cerebral arterial circle. Since the lateral branch of the vertebral artery also supplies the medulla oblongata, we included it in the vertebrobasilar contribution.

All measurements were undertaken on both right and left arteries. Missing values were from three samples, where the arterial branch had hemorrhaged, and the epoxy cast had leaked into the surrounding cavity. We tested all data for homogeneity using paired *t* test in MS Excel. None of the arteries showed significant differences between the left and right sides so we pooled the data for all samples. We calculated the percentage contribution of the carotid and vertebrobasilar systems and present data in Table 1. The areas of the brain supplied by the basilar artery and its branches were studied and described.

**Table 1:** Morphometric analysis: Mean Diameter (mm) of various cerebral arteries and testing for variation between the left and right pair.

**Arterial system**	**Artery**	**Mean diameter (mm)**	**Mean diameter and SD of left and right pair combined (mm)**	**Percentage contribution to the brain (%)**
Carotid	Right rostral cerebral artery	2.7	2.62 ± 0.33^**^	65.4%
	Left rostral cerebral artery	2.53		
	Right caudal communicating artery	2.68	2.63 ± 0.51	
	Left caudal communicating artery	2.55		
Vertebrobasilar	Right medullary segment of the basilar artery	2.32	2.38 ± 0.37	34.6%
	Left medullary segment of the basilar artery	2.45		
	Right lateral branch of the vertebral artery	1.24	1.26 ± 0.25	
	Left lateral branch of the vertebral artery	1.29		

** *Significant variation between the left and right pair of the same artery from paired t-test*.

The percentage contributions of the measured arteries were estimated as follow:

$$\begin{array}{l} \text{Percentage contribution of the artery in question}\\ =\frac{\text{Diameter of the artery in question X }100}{\text{The sum of diameters of all measured arteries}} \end{array}$$

## Results

### The Two Systems of Afferent Arteries to the Brain

Like all mammals, the dromedary brain is supplied with arterial blood from two major routes: the principal being that originating from the bicarotid trunk and the second being the vertebrobasilar system. In the former case the brachiocephalic trunk gives rise to the bicarotid trunk that at the level of the first rib divides into a left and a right common carotid artery. Each common carotid artery is found ventral to the cervical vertebrae and dorsolateral to the trachea giving off small branches to nearby structures and muscles.

At the level of the first cervical vertebra the common carotid artery gives sequential rise to the occipital artery, the internal carotid artery and then the condylar artery. It continues on as the external carotid artery that subsequently has branches that supply the rostral epidural rete mirabile. In summary, the carotid system of arteries supplies the brain through the internal carotid artery, occipital artery, rami rostrales of maxillary artery, external ophthalmic artery and middle meningeal artery. All of these arteries form the RERM from which the rostral cerebral arteries and the caudal communicating arteries originate and which in turn give off several branches to supply the brain.

The second source of arterial blood to the brain is the vertebrobasilar system that consists of a vertebral artery arising from the left and the right subclavian arteries. In dromedaries, the right subclavian artery arises from the brachiocephalic trunk while the left subclavian artery arises directly from the aortic arch.

**Figure 4:**
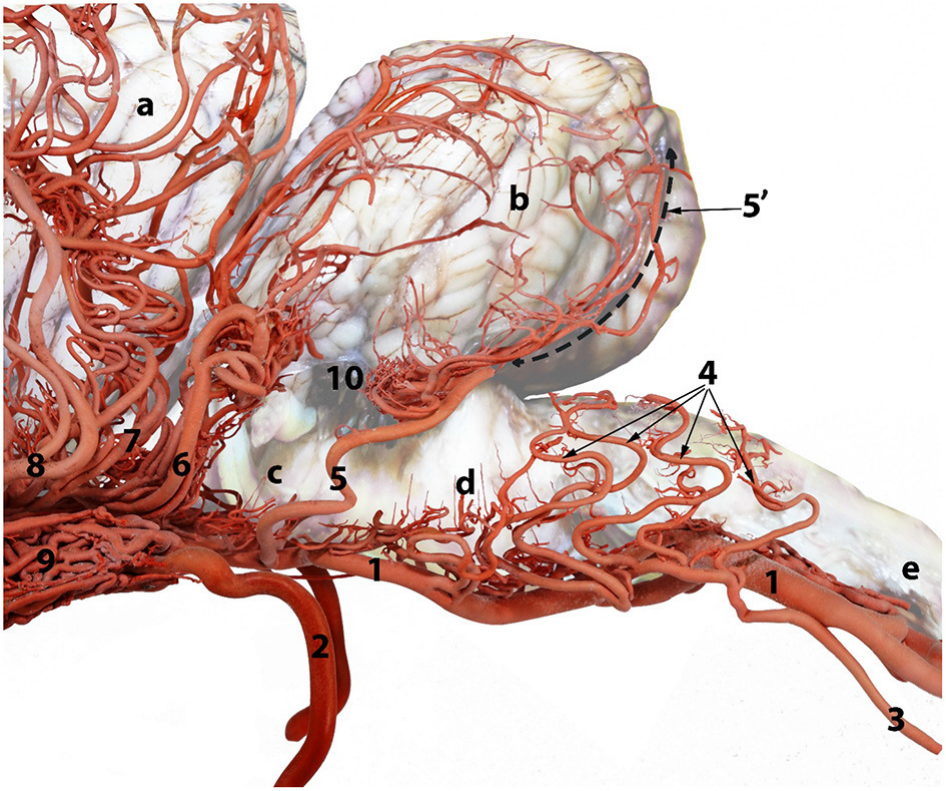
A composite photograph showing the arterial supply to the caudal regions of the dromedary brain (left lateral view). 1, basilar artery; 2, internal carotid artery; 3, lateral branch of the vertebral artery; 4, medullary branches; 5, caudal cerebellar artery; 5', dorsal arch of caudal cerebellar artery; 6, rostral cerebellar artery; 7, caudal choroidal artery; 8, caudal cerebral artery; 9, rostral epidural rete mirabile (RERM); 10, choroid plexus of the fourth ventricle; a, cerebrum; b, cerebellum; c, pons; d, medulla oblongata and e, spinal cord.

**Figure 5:**
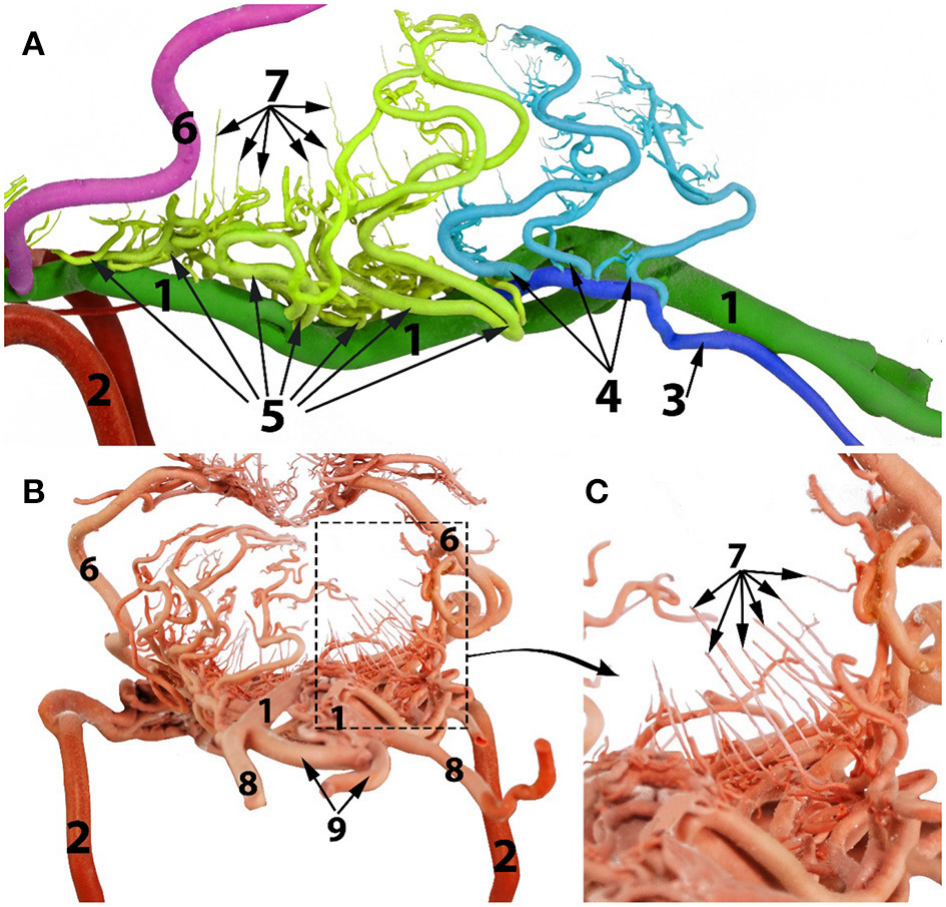
Arterial supply to the medulla oblongata of the dromedary brain. **(A)** left lateral view; **(B)** caudal view; **(C)** magnified part of the image **(B)** to show the straight vertical medullary branches. 1, basilar artery 2, internal carotid artery; 3, lateral branch of the vertebral artery; 4, medullary branches from the lateral branch of the vertebral artery; 5, medullary branches from the basilar artery; 6, caudal cerebellar artery; 7, median, paramedian and lateral medullary branches; 8, vertebral artery; 9, ventral spinal artery.

In dromedaries, the vertebrobasilar arterial system contributes 34.6% of the afferent arterial blood to the cerebrum, cerebellum and brain stem (Table 1). It is the principal supply to the cerebellum and the only source of arterial blood to the medulla oblongata and pons (Figures 4, 5).

### Vertebral Artery

#### Vertebral Artery Origin

In the dromedary, the aorta rises dorsally from the left ventricle of the heart at the level of the fourth rib to then curve cranially forming the brachiocephalic trunk that immediately gives rise to the left subclavian artery at the level of the third intercostal space. As the left subclavian artery runs cranially toward the thoracic inlet it gives rise to the left costocervical trunk and when level with the caudal border of the first rib it gives rise to the left vertebral artery. The right subclavian artery arises from the brachiocephalic trunk and gives rise to arterial branches in a similar manner to those of the left subclavian artery. Both vertebral arteries run forward on the longus colli muscle and run a short distance along the neck before entering their respective transverse foramen.

#### Vertebral Artery-Cervical Phase-Route

There is considerable variation in the vertebral artery's point of entry into the transverse canal. In most cases each vertebral artery passes between the middle and ventral scalenus muscles to enter the transverse foramen of seventh cervical vertebra (C7). In many samples only the right or the left transverse foramen of the 7th cervical vertebra was present. When this foramen was present, the vertebral artery entered the cervical vertebral canal through it. In cases where the transverse foramen of C7 was absent, the vertebral artery entered the cervical vertebral canal through the intervertebral foramen between the sixth and seventh cervical vertebrae.

**Figure 6:**
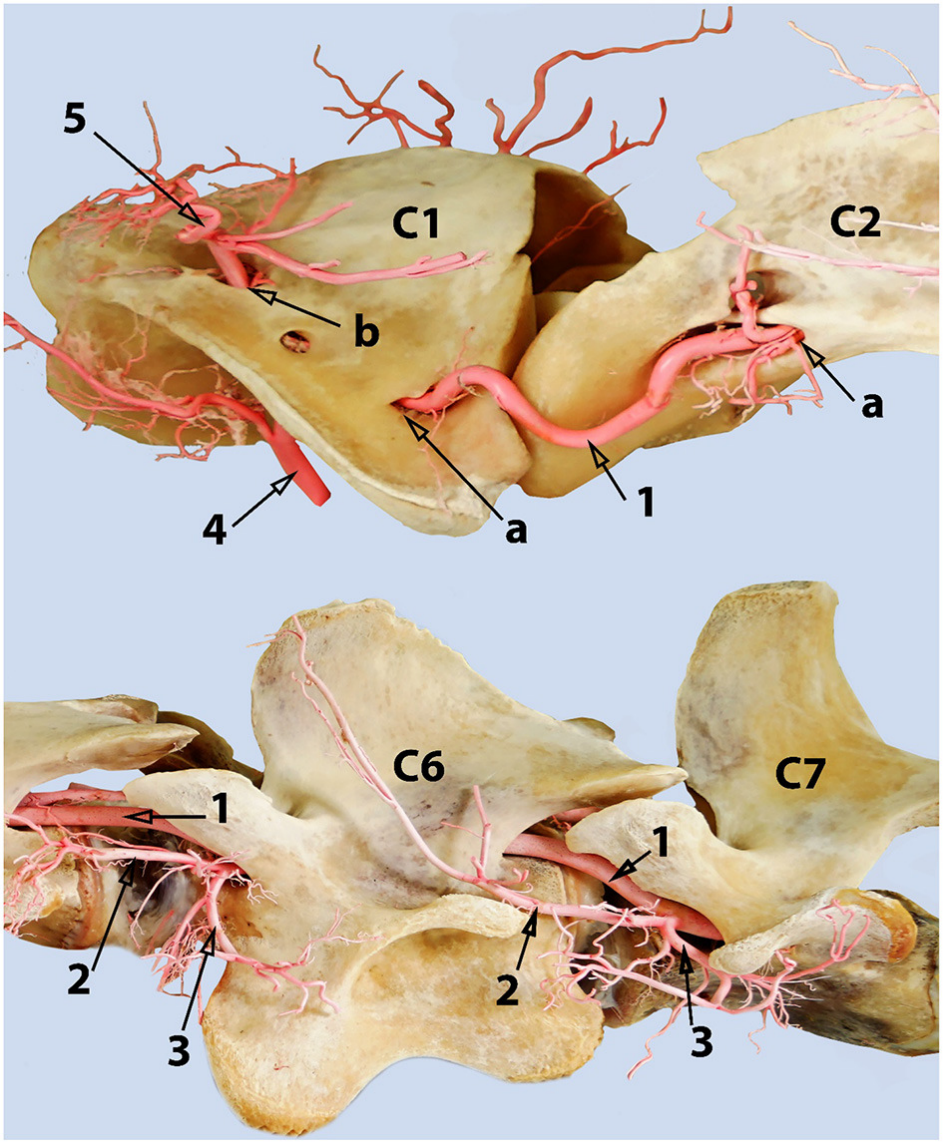
A composite photograph (left lateral view) showing the cervical phase-route of the vertebral artery. Where 1, vertebral artery; 2, dorsal muscular branch of the vertebral artery; 3, ventral muscular branch of the vertebral artery, 4, occipital artery; 5, muscular branches arising from the anastomosis between the vertebral and the occipital arteries; a, transverse foramen; b, alar foramen. The vertebral artery anastomoses with the occipital artery inside the alar canal. The left transverse foramen of the 7th cervical vertebra is present, and the vertebral artery enters the vertebral canal through it in this sample.

**Figure 7:**
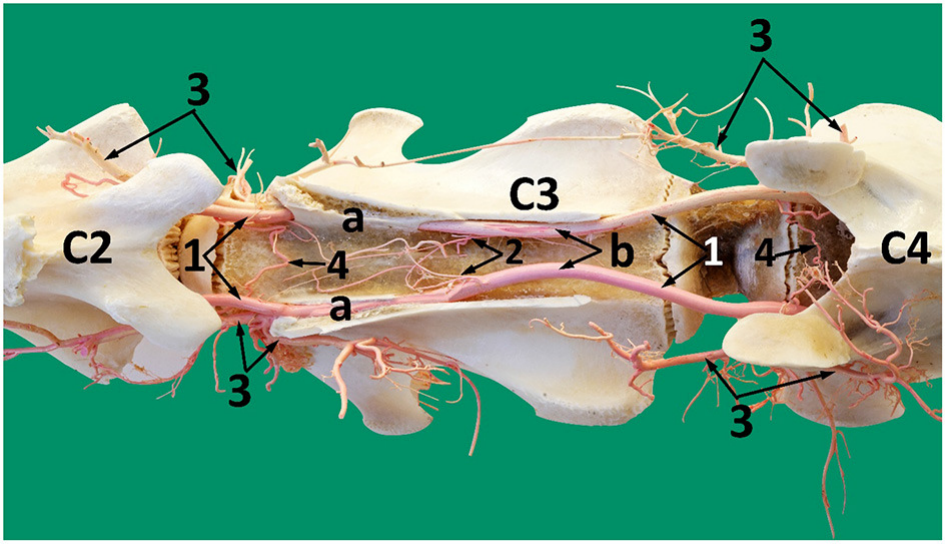
A photograph showing the route of the vertebral artery inside the vertebral canal (dorsal view). 1, vertebral artery; 2, spinal branches of the vertebral artery; 3, ventral and dorsal muscular branches of the vertebral artery, 4, tortuous shunt between the right and the left vertebral arteries; Note: The vertebral artery travels partly through the transverse canal (a) and partly through the vertebral canal (b).

Each vertebral artery traverses the length of the neck passing sequentially through the transverse canal and partly through the vertebral canal, accompanied by the vertebral vein and vertebral nerve (Figures 6, 7). After emerging from the transverse canal, as it continues in the vertebral canal the vertebral artery gives rise to prominent muscular branches. These muscular branches exit the vertebral canal at the intervertebral foramina and then immediately divide into dorsal and ventral branches mirroring the external routes of the dorsal and ventral branches of the cervical spinal nerves (Figures 6, 7).

**Figure 8:**
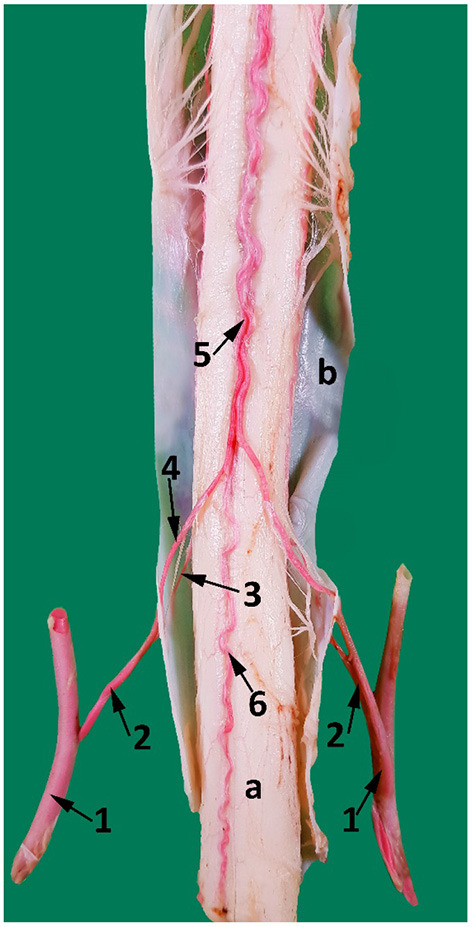
A ventral view of the spinal cord at the level between the third and the fourth cervical vertebrae. 1, vertebral artery; 2, spinal branch located between C3 and C4; 3, lateral spinal branch; 4, ventral spinal branch; 5, paired ventral spinal arteries that fuse to form a single ventral spinal artery cranially; 6. single ventral spinal artery; a, spinal cord; b, dura matter.

**Figure 9:**
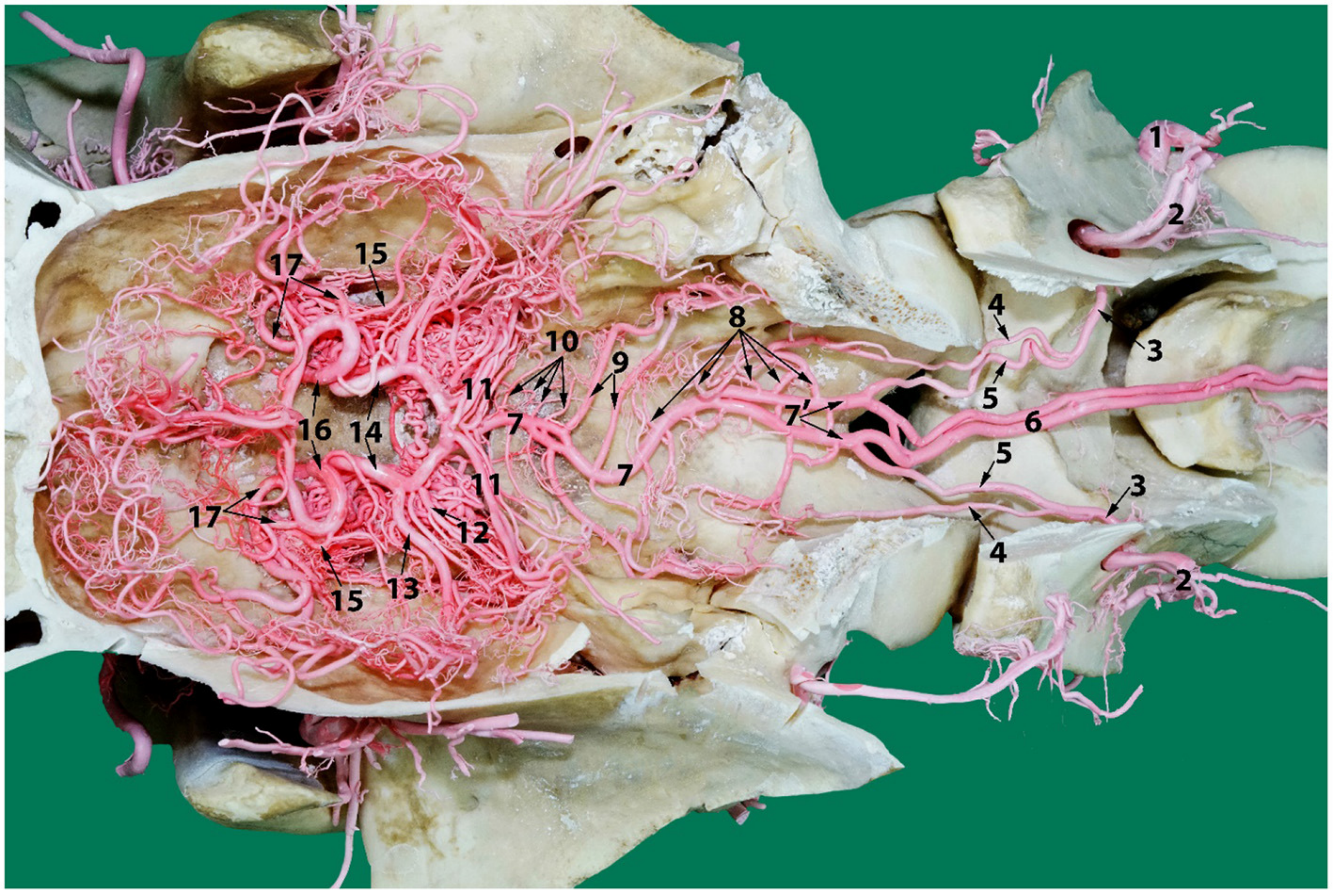
A photograph showing the arterial supply to the dromedary brain (dorsal view) lying on the ventral floor of the cranial cavity and of cervical vertebrae 1 and 2. Here; 1, vertebral artery; 2, dorsal muscular branch; 3, vertebral artery inside the vertebral canal; 4, lateral branch of the vertebral artery; 5, medial branch of the vertebral artery; 6, paired ventral spinal arteries; 7', right and left segments of the basilar artery; 7, basilar artery; 8, medullary branches; 9, caudal cerebellar artery; 10, pontine arteries; 11, rostral cerebellar arteries; 12, caudal choroidal artery; 13, caudal cerebral artery; 14, caudal communicating artery; 15, rostral choroidal artery; 16, rostral cerebral artery; 17, middle cerebral artery. Note that the RERM lies ventral to the cerebral arterial circle in the cavernous sinus at the base of the cranial cavity.

Within the vertebral canal each vertebral artery gives rise to a single spinal branch generally in the mid segment of each cervical vertebra (Figures 7, 8). Each spinal branch passes through the dura mater and arachnoid mater to enter the subarachnoid space where they bifurcate into two branches. The larger ventral branch traverses to the ventral median sulcus of the spinal cord to fuse with its opposite number. This fusion results in a single median ventral spinal artery. In some samples the two ventral branches do not fuse and run together cranially immediately below the ventral median fissure (Figures 8, 9). At each intervertebral foramen, the vertebral artery sends out segmental branches that also contribute to the ventral spinal artery. Of these contributors, those at the level of third/fourth and first vertebra are most prominent (Figures 7, 8).

The smaller lateral spinal branch follows the route of the dorsal nerve root to form a separate mesh of end arteries supplying the dorsal and lateral surface of the spinal cord (Figure 8). The vertebral artery also supplies thin nutrient branches to the vertebral bodies, especially near the intervertebral joints/discs.

#### Feeder Arteries From Vertebral Artery

During its course through the vertebral canal, prominent large muscular branches arise from the vertebral artery immediately after its exit from the transverse canal. At the level of the cranial vertebral notch, each muscular branch emerges from the intervertebral foramen before dividing into dorsal and ventral muscular branches that supply the surrounding neck muscles (Figure 6).

**Figure 10:**
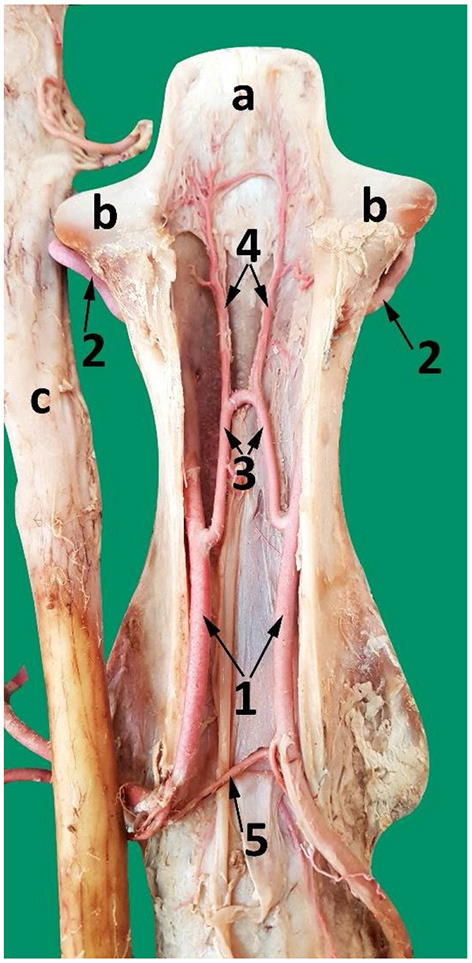
A dorsal view of the second cervical vertebra (axis) after removing the dorsal roof showing the course of the vertebral artery and its branches inside the axis. 1, vertebral artery inside the vertebral canal; 2, vertebral artery after exiting the transverse foramen of the axis; 3, communicating branch between the left and right vertebral arteries (apical arcade); 4, ascending arteries; 5, spinal branch; a, dens of the axis; b, cranial articular process; c, spinal cord reflected from the vertebral canal.

Another branch emerges from the vertebral artery close to the rostral edge of each cervical vertebra body. They join with their opposite pair to form a tortuous shunt (Figure 7). Smaller branches arise from this interconnection to enter the body of the cervical vertebrae. Some nutrient branches supply the bone and intervertebral disc. At the level of the middle of the second cervical vertebra (axis), a single branch emerges from each vertebral artery to anastomose with its fellow to form the apical arcade (Figure 10). The apical arcade sends out two ascending arteries that run cranially and when level with the cranial articular processes form an anastomotic arcade before sending out several small branches that fan out to the dorsal surface of the dens to supply the adjacent region including the structures of the atlantoaxial joint (Figure 10).

#### Vertebral Artery-Anastomosis and End Phase-Route

**Figure 11:**
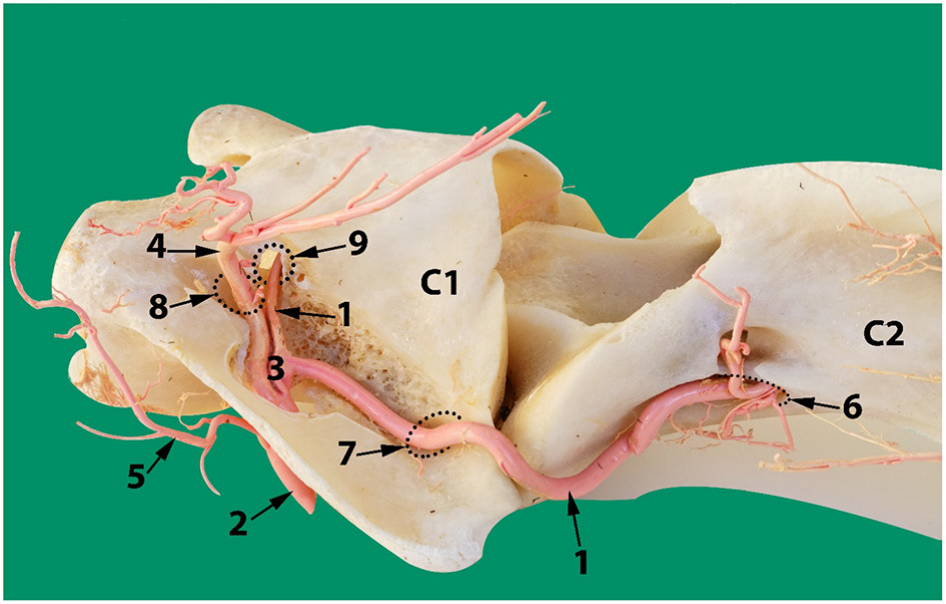
Dorsolateral view of the first two cervical vertebrae of the camel showing the route of the vertebral artery inside the transverse canal of the atlas and its anastomosis with the occipital artery inside the alar foramen. 1, vertebral artery; 2, occipital artery; 3, anastomosis between the vertebral artery and the occipital artery; 4, dorsal muscular branch; 5, ventral muscular branch; 6, ventral opening of the transverse foramen of the axis; 7, transverse foramen of the atlas; 8, dorsal opening of the alar foramen; 9, lateral vertebral foramen.

The occipital artery arises from the external carotid artery and runs dorsally in close proximity with the internal carotid artery. As it traverses toward the atlantal fossa, it gives off thick muscular branches to the surrounding neck muscles both before entering and after exiting the alar canal (Figures 6, 11).

The alar canal is a short canal traversing through the wing of atlas connecting its ventral and dorsal openings. The occipital artery enters through the ventral opening of the alar canal. The vertebral artery enters through the transverse foramen at the caudal border of the wing of the atlas and runs inside the transverse canal to meet and anastomose with the occipital artery inside the alar canal (Figures 6, 11).

After anastomosing with the occipital artery, the vertebral artery enters the vertebral canal via the lateral vertebral foramen (Figure 11). It then bifurcates into a medial and a lateral branch (Figure 9). Both branches run together outside the dura mater for a considerable distance before penetrating the dura mater. At the caudal border of the medulla oblongata, the medial branch travels medially to the ventral median fissure of the medulla oblongata. It meets and fuses with the ventral spinal artery of its side forming a basilar artery. Further cranially the left and right basilars fuse forming a single basilar artery (Figure 9).

The lateral branch of the vertebral artery travels rostrally up to the level of caudal cranial fossa before making a hairpin turn to join the basilar artery. As it does this it has several anastomotic branches with the medullary branches of the basilar artery (Figure 9).

### Basilar Artery

#### Basilar Artery Origin and Variations

Each vertebral artery fuses with its corresponding ventral spinal artery forming the right and left segments of the basilar artery which travel together for varying distances whilst giving rise to prominent branches to the medulla oblongata at the level of the hypoglossal nerve. The first branch at the caudal border of the medulla oblongata usually anastomoses with or is seen looped with the lateral branch of vertebral artery (Figures 9, 5).

Whilst the site of fusion of the right and left segments to form a single basilar artery varied, ranging from the level of the alar foramen through to the caudal border of pons, the most common point of fusion was midway along the length of the medulla oblongata. In three cases the two branches did not fuse but traveled together to the level of the cerebral arterial circle. In ten cases the basilar artery was seen to divide and rejoin at the mid-level of the medulla oblongata to form small ovoid rings (Figure 9).

The relative contribution of the ventral spinal artery and the vertebral artery to the formation of the basilar artery varied among camels. In most specimens the vertebral artery appeared to be the major contributor to the basilar artery. However, in four specimens the situation was reversed.

The route of the basilar artery is tortuous and this tortuosity is most prominent as it passes along the floor of the caudal cranial fossa. At this level it sends out numerous medullary branches that join the network of arteries in this area.

#### Basilar Artery Branches

In the medullary region the route of the basilar artery is highly tortuous and may have several bifurcations and reconnections (Figure 9). The basilar artery whether single or doubled gives rise to a series of sinuous medullary branches as it traverses the length of the medulla oblongata (Figure 9). In the caudal cranial fossa the basilar medullary branches communicate with the medullary branches of the lateral branch of the vertebral artery as well as with branches of the caudal cerebellar artery (Figures 4, 9, 5). This complex of arteries traverses over the curved body of the medulla to supply the lateral and dorsal aspect of the medulla oblongata (Figures 4, 5).

The basilar medullary arteries also give off numerous slender, vertically running median, paramedian and lateral medullary penetrating branches that project deeply into the medullary parenchyma to supply the depths of the median, paramedian and lateral regions of the medulla oblongata (Figures 4, 5).

**Figure 12:**
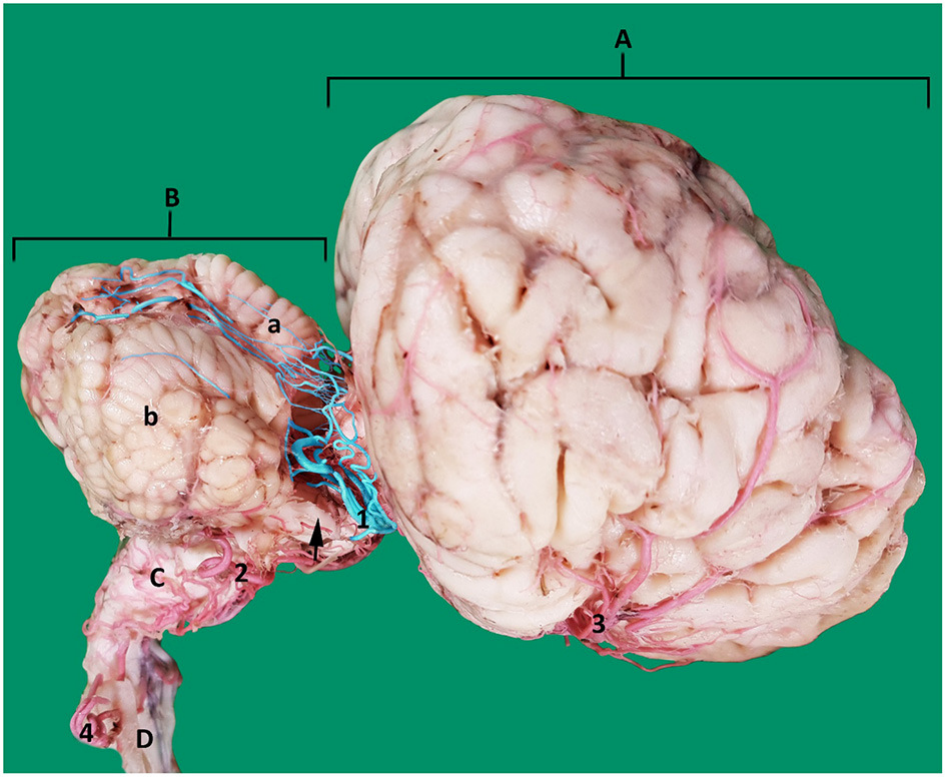
Lateral view of an arterial cast of a dromedary brain. A, cerebrum; B, cerebellum where a, vermis and b, right cerebellar hemisphere; C, medulla oblongata; D, spinal cord; 1, rostral cerebellar artery; 2, caudal cerebellar artery; 3, middle cerebral artery; 4, vertebral artery. Note how the rostral cerebellar artery (highlighted in blue) (1) crosses the root of the trigeminal nerve (arrowed) and cerebral peduncle before moving toward the groove between vermis and cerebellar hemisphere on the rostral surface of the cerebellum.

At the rostral end of the medulla oblongata and at the caudal border of the pons, two caudal cerebellar arteries originate, about 7 mm apart, from the basilar artery and run parallel to each other across the floor of the caudal cerebral fossa (Figure 9). From the rostral root of the caudal cerebellar artery a small branch artery, the labyrinthine artery, was observed to accompany the vestibulocochlear nerve toward the internal auditory meatus. The two roots of the caudal cerebellar artery run caudolaterally encircling the abducens nerve before fusing to form the main trunk of the caudal cerebellar artery. This travels dorsolaterally to the caudal cerebellar peduncle where it splits in two. The smaller branch runs caudal to the facial nerve to supply the lateral aspect of the cerebellum. The larger caudal branch arises from the dorsal aspect of the medulla to follow the groove between the vermis and the lateral cerebellar hemisphere (Figures 4, 12). As the artery climbs in a gentle curve medially it forms a dorsal arch over the caudal surface of the cerebellum (Figure 4). While doing so, it gives off branches to the caudoventral aspect of the cerebellum. These supply the choroid plexus of the fourth ventricle (Figure 4). It also anastomoses with medullary branches of the basilar artery to supply the medulla oblongata on the dorsal aspect of its cranial border. The caudal cerebellar artery also anastomoses with the rostral cerebellar artery and pontine arteries.

As the basilar artery continues anteriorly along its path it traverses ventrally to the pons where it gives off several pontine arteries. These thin, short vessels are characterized by being evenly spaced and running parallel to each other. The pontine arteries have many anastomoses with the caudal cerebellar arteries and with the rostral cerebellar arteries.

The ventrolateral aspect of the medulla oblongata and pons is supplied mainly by the median, paramedian and lateral penetrating branches running perpendicularly from the medullary and pontine arteries to pass directly into the depths of the medulla and pons (Figures 4, 5).

At the rostral end of the pons, immediately before it anastomoses with the caudal communicating artery, the basilar artery gives off several large rostral cerebellar branches that run in a caudolateral direction in the transverse cerebral fissure to supply the rostrodorsal aspect of the cerebellum (Figures 4, 9).

The rostral cerebellar artery is the most variable artery of the system in terms of its size, number of contributors and points of origin. There are usually 4–6 arteries arising mostly from the basilar artery. The primary identifying factor of the rostral cerebellar artery is its caudolateral direction, that it crosses the root of the trigeminal nerve and cerebral peduncle before moving toward the transverse cerebral fissure and then to traverse through the groove between the vermis and the lateral cerebellar hemisphere over the rostral surface of the cerebellum. It supplies the rostral and caudal colliculi of the mesencephalon as well as the base and rostrodorsal surface of cerebellum before anastomosing with the end arteries of caudal cerebellar artery (Figures 4, 12).

### Blood Flow Direction in the Vertebral and Basilar Arteries

Transcranial color doppler ultrasonography in the sagittal plane identified the vertebral artery in the neck at the level of the first to the second cervical vertebrae. Within it the blood flow was toward the brain (Figure 1C).

The basilar artery was identified in the ventral median fissure of the medulla oblongata in both transversal and sagittal planes. Its direction of blood flow was toward the brain (Figures 1A,B). The same result was obtained in ventral recumbency as well as when in a standing position.

### Percentage Contribution of Carotid and Vertebrobasilar Systems

In the current study (*N* = 28), the mean diameter ± SD of the rostral cerebral artery was 2.62 ± 0.33 mm, while it was 2.63 ± 0.51 mm for the caudal communicating artery. In the vertebrobasilar system, the mean diameter ± SD of the basilar artery was 2.38 ± 0.37 mm, and that of the lateral branch of the vertebral artery was 1.26 ± 0.25 mm (Table 1). Our measurements showed in the dromedary that the percentage contribution of the carotid system to the brain was 65.4% while the percentage contribution of the vertebrobasilar system was 34.6%.

## Discussion

In our study using transcranial color doppler ultrasonography we proved that the direction of blood flow in the vertebral and the basilar arteries was toward the cerebral arterial circle. We found that the vertebrobasilar system exclusively supplies the medulla oblongata, pons and cerebellum and were able to demonstrate that the vertebrobasilar arterial system contributes a significant proportion (34.6%) of the arterial blood supply to the brain of the dromedary.

### Direction of Flow of Basilar Artery

The vertebrobasilar system is considered a significant source of afferent blood in dromedaries (17, 22). However, the vertebrobasilar system has a less important role in the arterial blood supply to the brain in cattle and sheep because the blood flow to the brain through the carotids is significantly higher than that of the vertebral arteries (10, 11, 14, 19, 24, 25). In sheep the caudal communicating arteries anastomose forming the basilar artery where the direction of blood flow is caudal, away from the cerebral arterial circle. While moving caudally, the basilar sends out branches supplying to the pons and medulla oblongata (12–16).

Several research groups have described the basilar artery of artiodactyls such as the goat, sheep and deer to taper as it runs caudally in the caudal cerebral fossa to later continue as the ventral spinal artery (3, 6, 15, 19, 26).

In dromedaries, however, Kanan reported that the diameter of the basilar artery decreases as it traverses toward the cranium possibly indicating that the arterial blood flow is toward the arterial circle (27). This is supported by our ultrasound investigations where we found that the basilar blood flow is toward the cerebral arterial circle.

In our study, we describe the basilar artery supplying the cerebellum, medulla oblongata and the pons directly before it joins the cerebral arterial circle. The basilar artery gives rise, sequentially from the caudal aspect of the medulla, to the medullary arteries, pontine arteries, rostral cerebellar arteries and caudal cerebellar arteries. The direction of blood flow in the basilar artery, toward the cerebral arterial circle or away from it, has major effects on time of death following halal slaughter.

### Ventral Spinal Artery Role as Contributor

In our study, the basilar artery was seen to be a direct continuation of the ventral spinal artery with the majority of its blood flow being delivered through anastomoses with the vertebral artery. This vascular architecture is similar to the contributory role of vertebral artery in the dog reported in recent times by Salomon (28, 29). In our study, the vertebral artery is seen contributing directly and indirectly to the basilar artery at multiple locations: Indirectly by forming the ventral spinal artery at the level between the third and the fourth cervical vertebrae, which contributes later to the basilar artery, and directly at the level of the first cervical vertebra and at the caudal cranial fossa (Figure 9).

These contributions run counter to Kanan's suggestion that the ventral spinal artery is an end branch of the vertebral artery in the dromedary (27).

The direction of blood flow identified by our ultrasound investigations as well as the narrowing in diameter of the basilar artery toward the brain support the idea that the ventral spinal artery actually continues cranially as the basilar artery or even contributes significantly to the basilar artery. Our study supports the idea that the ventral spinal artery fuses with the medial branch of the vertebral artery and continues cranially as the basilar artery with a substantial quantity of blood being contributed by the vertebral artery as well as the occipital artery, at the level of alar foramen (28, 29). Similar observations of the ventral spinal artery fusing with the vertebral artery have been made in the dog, cat and rabbit (30). The same author has observed the absence of this pattern in rats, mice and guinea pigs, where small branches from the vertebral artery flows caudally to form the ventral spinal artery.

### Estimating Percentage Contribution of Vertebrobasilar System

To date, Ocal et al. (23) and Ocal et al. (10) have been the only researchers to undertake a quantitative study of the dromedary's cerebral vasculature. They reported the percentage contribution of the maxillary artery, internal carotid artery and external ophthalmic artery to the RERM as 76.41, 13.33, and 10.26, respectively (23). Subsequently they measured the diameter of the arteries contributing to the cerebral arterial circle namely the rostral cerebral artery, the caudal communicating artery and the basilar artery (10). However, their estimates regarding the vertebrobasilar system did not include the lateral branch of the vertebral artery nor consider the initial branches from the unfused basilar artery which play an important role in supplying the medulla oblongata.

Ocal et al. did not calculate or estimate the percentage of the vertebrobasilar and the carotid system contributions to the cerebral arterial circle in their study. By using Ocal's data, with regards to the measured diameters of the arteries, we calculated the percentage of blood contributed by these arteries. These calculations indicated that the vertebrobasilar and the carotid system contributions to the cerebral arterial circle are 21 and 79%, respectively. The vertebrobasilar contribution appears to be underestimated because Ocal did not include the lateral branch of the vertebral artery as well as the initial branches from the unfused basilar artery supplying the medulla oblongata. In this study, we measured the basilar artery at the level after the fusion of the vertebral and ventral spinal arteries before sending medullary branches to the medulla oblongata (Figure 3) and the percentage contribution of the carotid system to the brain was 65.4% while the percentage contribution of the vertebrobasilar system was 34.6%.

When we estimated the percentage contribution using our measurements based on the arterial location used by Ocal et al. (10), we had a similar vertebrobasilar contribution of 21% to the cerebral arterial circle. This validates our data that yielded our estimate.

### Double Root of Caudal Cerebellar Artery and Labyrinthine Artery

In our study the caudal cerebellar artery in most of our samples had two roots that arose from the basilar artery about 7 mm apart (Figures 9, 12). A double root of the caudal cerebellar artery has been reported in camelids, chinchilla and deer (21, 26, 27, 31). Contrastingly in cattle, goats and dogs the caudal cerebellar artery has been reported as having a single root of origin (3, 25). In our study the two caudal cerebellar arteries both ran laterally. At the lateral border of the medulla, they fused with each other to form a common artery before continuing to supply the caudal aspect of the cerebellum (Figures 9, 12).

In our study, we saw the labyrinthine artery as a small branch from the rostral root of the caudal cerebellar artery that follows the vestibulocochlear nerve toward the internal auditory meatus. This result is consistent with the study of Kanan (27) who documented this artery supplying the internal ear in the camel.

## Data Availability Statement

The raw data supporting the conclusions of this article will be made available by the authors, without undue reservation.

## Ethics Statement

The animal study was reviewed and approved by Animal Research Ethics Committee at the United Arab Emirates University.

## Author Contributions

AA: study design/conception, anatomical dissection, analysis/acquisition of data, photography and photo editing, drafting of the manuscript, and revision of the manuscript. PM: study design, anatomical dissection, analysis/acquisition of data, drafting of the manuscript, and revision of the manuscript. AAD: specimen collection, specimen preparation. MQ and TS: correction and revision of manuscript. MH: study conception, consultation of used casting materials. KR: guidance, correction and revision of manuscript. All authors contributed to the article and approved the submitted version.

## Conflict of Interest

The authors declare that the research was conducted in the absence of any commercial or financial relationships that could be construed as a potential conflict of interest.
